# John Langdon Down (1828 – 1896)

**Published:** 2016-06-27

**Authors:** J Van Robays

**Affiliations:** Department of Pathology, ZOL, Campus St Jan, Schiepse Bos 6, 3600 Genk, Belgium.

**Figure:**
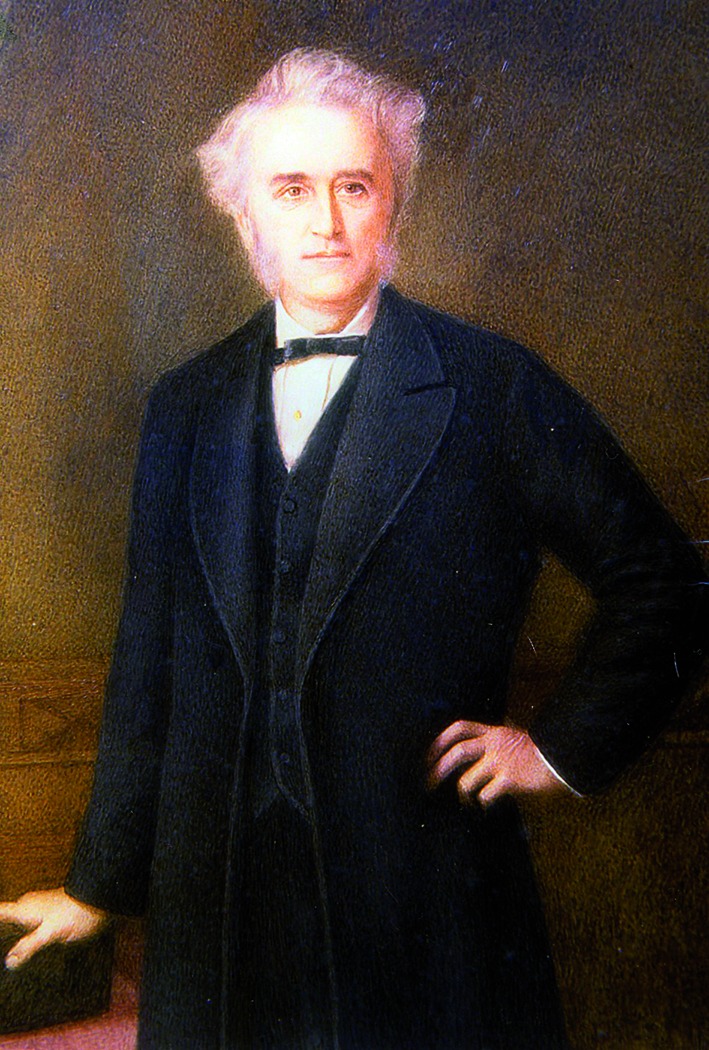


When John Down was 18 years old, he had some kind of mystical experience. He met a girl that appeared so peculiar, that he felt sorry for her and, consciously or unconsciously, would spend his whole life searching for this phenomenon. He decided to study Medicine, became the director of the largest “Asylum for Idiots” in England and wrote articles and books about the thing that fascinated him so. Anthropometrically and photographically, he delineated a well-defined group of mentally disabled individuals, whose members all resembled the little girl very well. He called them, in concordance with the ethnical insights of the then famous dr. Blumenbach, mongoloid “idiots”. Today they bear his own – more politically correct – name: syndrome of Down.

## The mystical experience

On a sunny summer’s day in 1846, John was taking a stroll with his family in the fields around Devon. All of a sudden, dark clouds appeared and it started to pour down. The family started running and looked for shelter in a nearby farm. There, they were offered a cup of tea by the most odd-looking girl. John had never seen such a face. The young girl didn’t say a word, didn’t laugh and looked unhappy. John wandered whether she might have a disease. And if so, which disease would that be? And if anyone could do anything about it?

Up until then, he had worked in his father’s grocery store/pharmacy – against his liking. After this strange encounter, he decided to become a doctor, but this was against his father’s will. Only after his father had died in 1853, John applied to the “Medical School of the London Hospital”. The registration fee wasn’t cheap, but he was able to stay for free in his sister’s house, who lived in London with her husband Philip Crellin. One day Philip’s sister, Mary Crellin, came to visit and John had another mystical experience. He fell madly in love. Mary and John got married in 1860.

## Earlswood

John had already proven to be a brilliant student. during his college years Even now, in the London Hospital, he excelled in erudition and diligence. His last year, he concluded with a gold medal in Medicine, Surgery and Obstetrics. On top of that, he received the summa cum laude medal of the best student of his year. It was therefore with consternation that his professors, who had wished a beautiful academic career for him, found out he wanted to work in the lunatic asylum. This “Asylum for Idiots” was situated in Earlswood, in the county of Surrey. With its 400 “madmen” and “idiots”, it was one of the largest centres in England. Once founded in an act of charity by vicar Andrew Reed with the support of wealthy Lords, it had run into serious trouble. The “Lunacy Commission”, which had to judge the welfare of the mentally ill since the Lunacy Act of 1845, was infuriated by what they encountered in the Earlswood Asylum. The children were housed in rooms of fifteen to twenty. All kinds of things were forbidden. Corporal punishment was rife. Hygiene was inadequate and mortality was high, mainly from typhus and tuberculosis. The Lunacy Commission opened up a new position as Chief Physician, and the recently graduated John Down turned out to be the best candidate. Down might not have had any experience with the mentally challenged, but he was sociable, inquiring and was able to inspire others. Another factor that played an important role, was his strong Christian conviction.

**Fig. 2:**
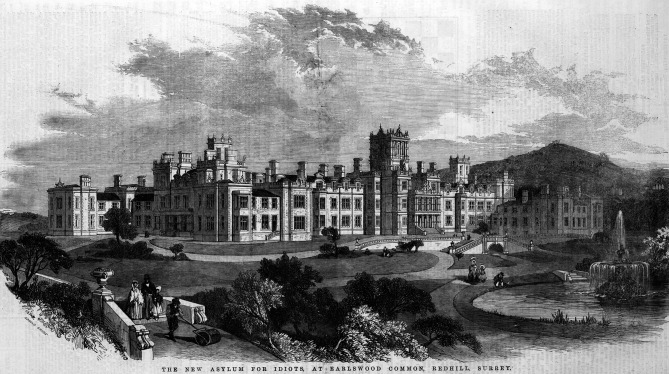
— Royal Earlwood Asylum of Idiots

## A fresh wind

John improved the policy of the Asylum in every respect. The board was recast, new staff was hired and hygiene became the top priority. He insisted that all the inhabitants of the institute ate with knife and fork. He prohibited all punishments. Good behaviour was rewarded and bad behaviour was condoned as much as possible. Children who were still bedwetting, were woken up at night to pee. To keep the inhabitants busy in a useful and pleasant way, John and Mary organized the most diverse activities. There was a lot of attention for diction and the mentally disabled were taught a new craft or hobby. Under the new policy, the Earlswood Asylum became an exemplary institute. A laudatory article appeared in the medical journal The Lancet, which made Earlswood world famous.

Asides from improving the physical and psycho-social conditions, John was also engaged in scientific research. In his era, not a lot was known about the causes of imbecility and idiocy. In Germany, Johann Friedrich Blumenbach had written the book “Uber die natürlichen Verschiedenheiten im Menschengeschlechte” and John was reading it in an English translation. By applying comparative anatomy and skull measurement, Blumenbach classified people into five races. The Caucasian race was white, the Mongolian yellow, the Malay brown, the Ethiopian black and the American red. Blumenbachs division was quite popular in those days, and John Down tried to use it to classify his Earlswood guests. To keep things clear and facilitate classification, he took a photograph of all of them. Not just a “mug shot” of their face, left profile and right profile; John Down had them pose in their nicest gown or suit, and in a flattering posture. The over 200 photographs which have remained – photography was still in its infancy – make up today’s biggest collection of clinical photography from the Victorian era.

When everyone had been photographed, measured and divided into ethnic groups, John Down published an article in the London Hospital Reports: “Ethnic Classifications of idiots”. The lion’s share of his attention went to what he called: “The great Mongolian family”. They were most numerously represented in Earlswood: “A very large number of congenital idiots are typical Mongols. So marked is this that, when placed side by side, it is difficult to believe the specimens compared are not children of the same parents.”

In “Mental affections of childhood and youth”, which was published later on, Down gave a full description of the external, innate features of a “mongoloid idiot”. The round face with oblique eyes, the flat nape, the short, bristly hair, the thin eyebrows, the small pug nose and the thick, cleaved tongue. Later on, the typical transverse palmar crease was added to this list. Down assumed parental tuberculosis might be the cause of this condition.

## Resignation in Earlswood

Mary Down did a lot of volunteering in Earlswood and was sort of the Florence Nightingale of the mentally disabled. She taught the children and was very creative in organizing recreation and amusement. She was also artistically and musically gifted and she taught a few “Mongols” to play short pieces on the piano. She organized chants and concerts as well. With their renovations and modern ideas, John and Mary were way ahead of their time, and evidently this was not to the liking of the high Lords of Earlswood. When John asked if his wife could be put on the payroll of the Asylum for the many hours she spent there, this was curtly refused. Women volunteered. And volunteering was free of charge. In time, the relationship between Down and the old Lords of Earlswood deteriorated, and when they refused to give him financial support to display the handicrafts of the disabled in an exhibition in Paris, he resigned in 1868.

## Normansfield

After this resignation, he could have become fulltime physician-surgeon in London again. After all, he was still a member of the Royal College of Surgeons and had retained a small private practice in London during all those years. But John got into new plans. In Kings Road, Hampton Wick, he had seen a beautiful, big, white house with a large adjacent domain. He dreamt of converting this White House in a home for the mentally disabled. To keep everything affordable, he decided to start with a dozen of children of rich Lords. He knew that the higher classer liked to pay to have their mongols, which were also born in their families, “placed” in a facility. This usually came down to “dumping” them for life, but this wasn’t how Down intended it. The families which wanted to place their mentally disabled child, had to agree to have the child privately educated, so they would be able to lead a qualitative life. But when John made the final calculations, both the revenues of the rent and the private lessons weren’t sufficient to finance the whole project. Luckily, he knew a lawyer who offered him a mortgage loan. Out of gratitude for the loyal proposal of Normal Wilkinson, John named the house Normansfield. In 1868, 19 children of bankers, physicians, high army officers and nobility were admitted, and it became an immediate success. The majestic mansion was equipped according to the highest standard of that time, both in comfort and hygiene. Everyone who visited it, spoke highly of it. In no time, the demand exceeded the offer and Down decided to expand the house and the domain. Apart from new living spaces and schoolrooms, a farm with stables, piggeries and a vegetable garden were constructed. The majority of the physicians of his era thought the inhabitants of a lunatic asylum were unable to learn anything. John wanted to prove the contrary. He had noticed that his mongols were certainly able to be raised properly. They were very good, even excellent, at imitating behaviour. This aptitude to imitate could be addressed to teach them all kinds of things. John and Mary taught them how to ride a horse, clean the stables, grow vegetables and fruit, collect eggs and milk the cows. In the workshops, various crafts were taught. Weaving and making puppets for the puppet play were the most popular activities. Here Down learned that when children with a similar talent are put in the same group, their learning improves. Probably because there is less pressure of competition. Down also found that shopping is very useful in social training programs. And to not lose sight of the artistic component, a small theatre was added to the building complex. It served primarily to stage little plays, but was also used for concerts, to grace parties and to practice chants for the Sunday High Mass. The richly varied educational concept of John Down was unique in its kind and in the whole of England. It was also valued accordingly, considering the numbers. The number of inhabitants of Normansfield increased from 106 in 1876, to 160 in 1896.

**Fig. 3:**
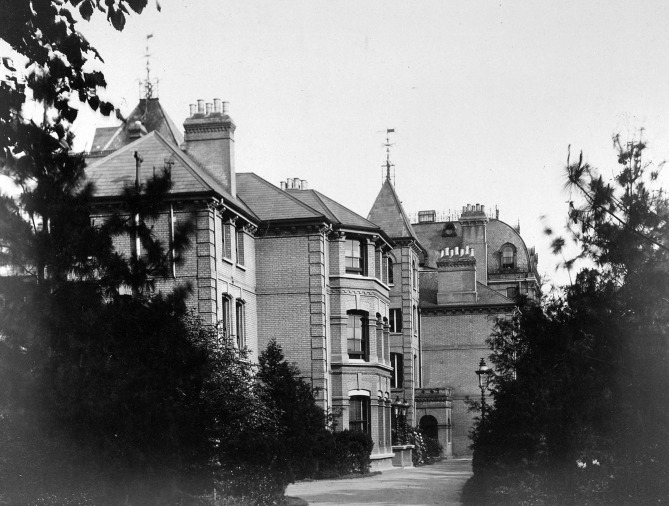
— Normansfield

Unexpectedly to many, John Langdon Down died at the age of 67 years in the fall of 1896. When the black veiled horses pulled the black carriage through the streets of Hampton Wick, all shops closed their doors. All curtains were lowered and at the side of the road, a crowd of people stood in silent sorrow. John Down was cremated and his ashes were kept in Normansfield. After his wife had died as well, their ashes were scattered together.

**Fig. 4a:**
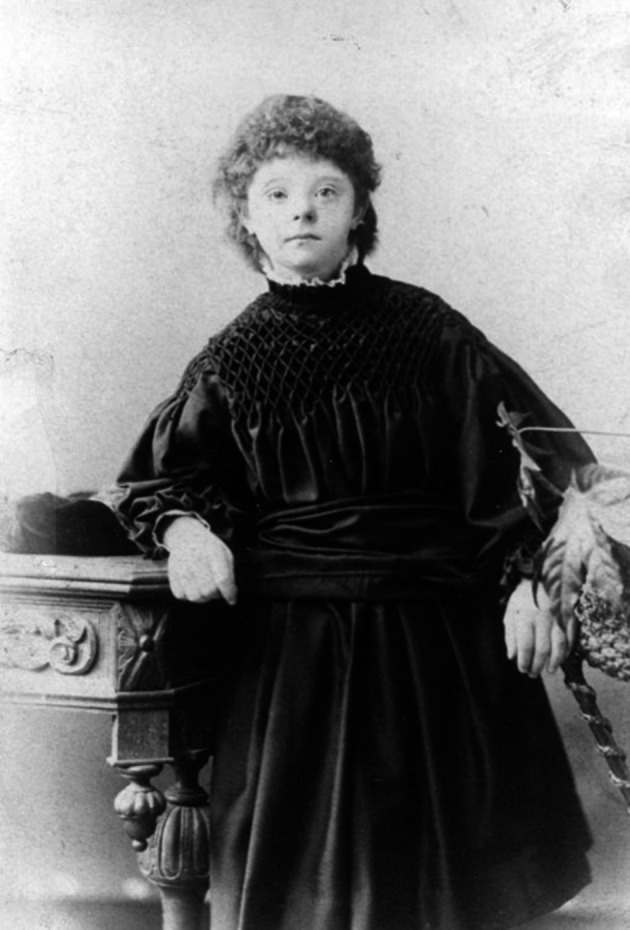
— Patient Flo Thornton – Age 20

**Fig. 4b:**
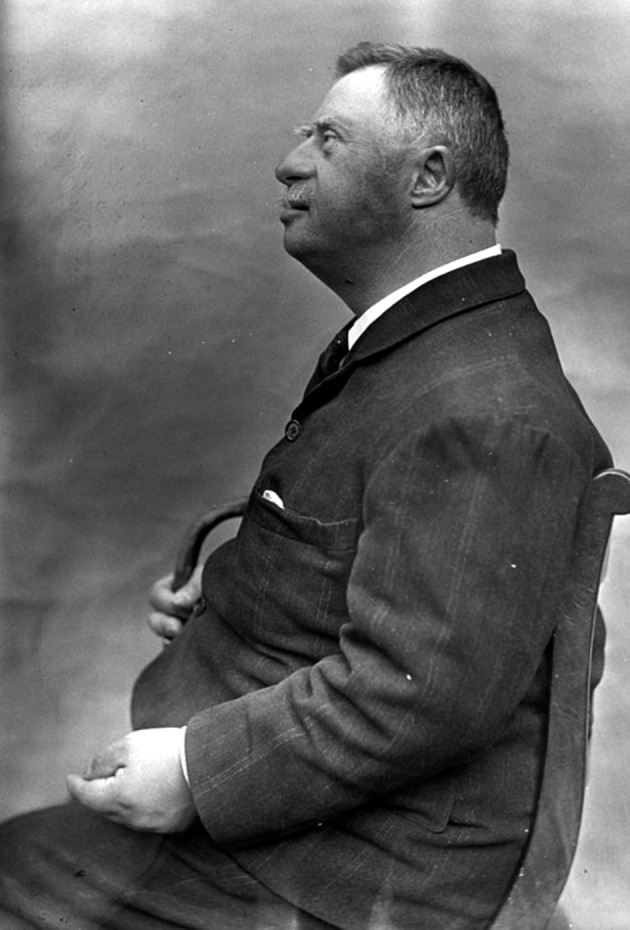
— Patient D Hillyer

**Fig. 4c:**
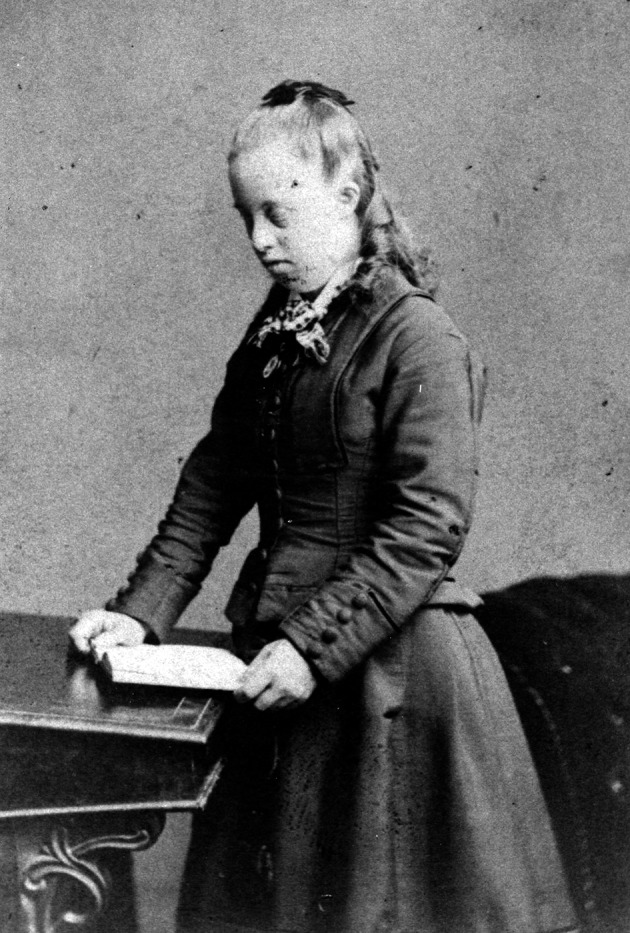
— Patient Mary Arnott – Age 19

**Fig. 5:**
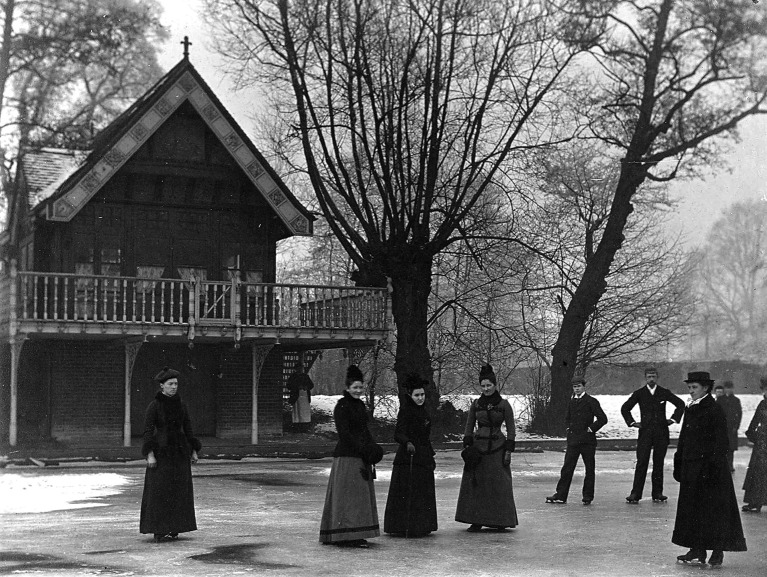
— Winter at the boathouse in Normansfield

## From idiot to trisomy

In the western world “mongolism” remained an accepted term until 1960. In 1961, a group of genetic experts wrote an open letter to the medical journal The Lancet. An increasing amount of Chinese and Japanese researchers, not to mention the inhabitants of Mongolia, found the association between Mongol and idiot ludicrous. Moreover, it was scientifically out-dated. Two years prior, it was discovered that the condition was actually due to an extra chromosome 21. Besides, trisomy 21 didn’t have any association with the normal Asian genes. Therefore, the writers of the letter insisted to completely ban the racist term “mongolism” and to propose an alternative. Any suggestion was welcome.

**Fig. 6:**
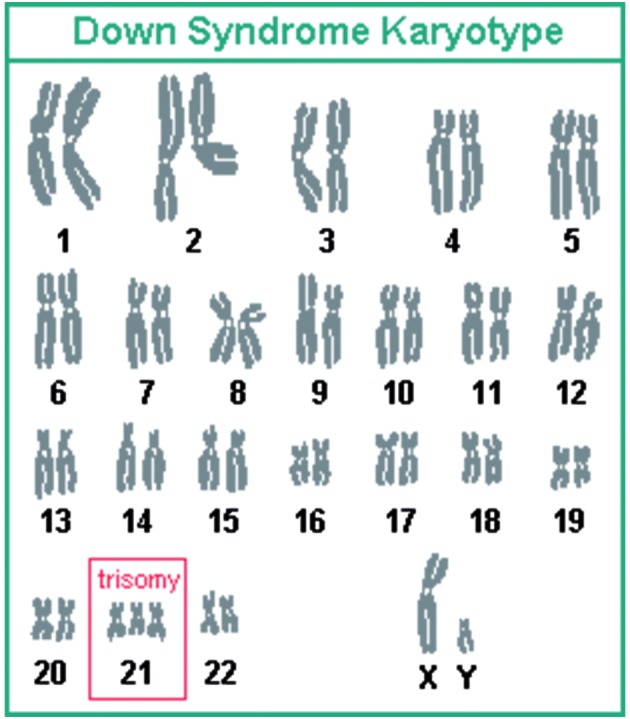
— Trisomy 21: Down syndrome karytype

One of the signatories of the letter approached John Langdon Down’s grandson, who at that time was still the head of Normansfield. He asked Norman (!) Langdon Down if his family name could be used to indicate the syndrome his grandfather had described so outstandingly. This was allowed, and the World Health Organization confirmed the eponym in 1965.

**Fig. 7:**
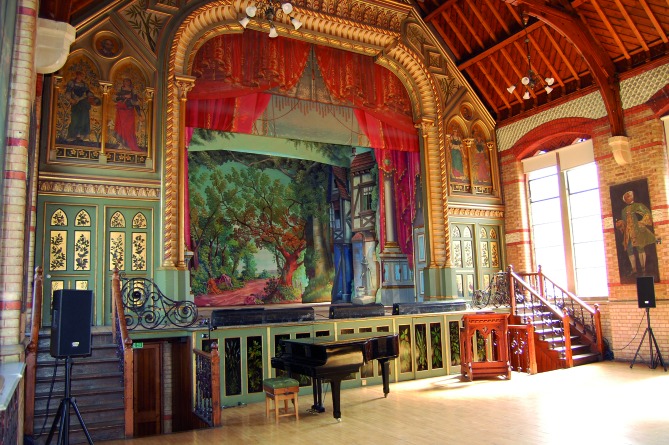
— Normansfield Theatre

